# Genetic risk score (GRS) constructed from polymorphisms in the *PON1*, *IL-6*, *ITGB3*, and *ALDH2* genes is associated with the risk of coronary artery disease in Pakistani subjects

**DOI:** 10.1186/s12944-018-0874-6

**Published:** 2018-09-27

**Authors:** NA Shabana, Sana Ashiq, Anam Ijaz, Fizah Khalid, Istabsar ul Saadat, Kahkashan Khan, Sumbal Sarwar, Saleem Ullah Shahid

**Affiliations:** Department of Microbiology and Molecular Genetics, University of the Punjab, Lahore, Pakistan

**Keywords:** Coronary artery disease, Polymorphism, Pakistan

## Abstract

**Background:**

Coronary artery disease (CAD) is a major killer in today’s world. Pakistan is also affected by this non-communicable disease like other countries. It is a multifactorial disease and is influenced by many gene-gene and gene-environment interactions.

**Methods:**

A total of 623 (219 controls, 404 cases) Pakistani subjects were genotyped for four SNPs, rs662 (*PON1*), rs5918 (*ITGB3*), rs671 (*ALDH2*), rs1800795 (*IL-6*) by PCR-RFLP. Various anthropometric parameters were noted and serum lipid profile was measured using commercially available kits. Statistical analysis was done by SPSS version 22. A Genetic Risk Score (GRS) was calculated from individual SNPs. The association of the SNPs and the GRS with CAD was checked using logistic regression.

**Results:**

The results showed that the risk allele frequencies of all variants were higher in the cases than the controls, however the difference was not statistically significant association (*p* > 0.0125). The mean GRS in the controls was 3.99 ± 1.42 and in cases, it was 4.29 ± 1.39, the difference between the groups was significant (*p* = 0.0109). logistic regression of individual SNPs and GRS with the CAD showed that independent SNPs were not significantly associated with the CAD however, the GRS had a strong association (*p* = 1.4 × 10^− 4^). The subjects were divided into three groups based on GRS (Gp 1 with GRS 0–2, Gp 2 with GRS 3–5 and Gp 3 with GRS 6–8). The analysis of the effect of the individual SNPs and GRS groups on different lipid profile parameters revealed no significant association of any of the tested SNPs with any lipid parameter, however, the GRS groups showed marginally significant for TC and highly significant association for TG, LDL-c and HDL-c.

**Conclusion:**

In conclusion, use of a GRS can provide better information than individual SNPs. The larger the number of the SNPs included in the analysis, the better would be the risk prediction.

## Background

Cardiovascular diseases (CVDs) are disorders in the two systems, the heart and the circulatory system. Among the group of cardiovascular disorders, coronary artery disease (CAD) is the most frequent [1]. The constriction of blood vessels due to atherosclerosis, leads to the poor blood supply to the heart [2]. In the developing countries its prevalence ranked the highest [1]. The increasing prevalence of coronary heart disease (CHD) in the South Asian countries poses a threat and huge burden to healthcare. The major reason of this increase was the adoption of modern lifestyle which also increases the risk of metabolic syndrome [3]. According to the World Health Organization (WHO) report from Global Burden of Disease (GBD), cardiovascular diseases are responsible for 31% of all global deaths. The mortality rate due to coronary artery disease alone in the year 2012 was 7.4 million while the total deaths due to cardiovascular disease was 17.5 million [4]. In Pakistan, one in every four adults suffered from coronary artery disease [5].

Coronary artery disease is a multifactorial disorder involving a complex interaction between environmental and genetic factors [6]. The modifiable and non modifiable risk factors are two broad categories of conventional risk factors [7]. Smoking, obesity, diabetes mellitus, hypertension, dyslipidemia, stress, depression and sedentary lifestyle are the modifiable risk factors while gender, age and family history are the non-modifiable risk factors [8]. We selected four SNPs from different genes. The rs662 SNP is located in exon 6 of paraoxanase 1 (*PON1*) gene which results in the substitution of A to G (arginine (R) at the place of glutamine (Q) in the protein) [9]. This single nucleotide polymorphism affects the catalytic activity for different substrates hydrolysis [10]. The change in catalytic activity is substrate dependent as Q allele hydrolyzes soman, diazoxon and sarin rapidly while R allele hydrolyzes paraoxon more efficiently [11]. The R allele carriers are more susceptible to cardiovascular disorders as Q192 is more effective in inhibiting oxidation of low density lipoprotein cholesterol as compared to R192 isoform [10]. The second selected SNP is rs5918 from integrin beta 3 (*ITGB3*) gene, present on chromosome 17 long (q) arm at 21.32 position. The product of *ITGB3* gene is a protein known as integrin beta III. It is also known as platelet glycoprotein IIIa, GP3A, GPIIIa or antigen CD61. A single base change in the exon 2 of this gene (C to T) results substitution of leucine (PlA1) amino acid for proline amino acid (PlA2) [12]. This polymorphism results in the different spatial orientation and conformational change in protein in fibrinogen-binding region [12]. It is suggested that this polymorphism has an important role in the progression coronary artery disease and coronary thrombosis [13] because a key event in acute coronary syndrome is the platelet aggregation with thrombus formation [14]. The third SNP was rs671 in the aldehyde dehydrogenase 2 (*ALDH2*) gene located on chromosome 12q24.2 [15]. Enzyme activity of *ALDH2* reduces due to rs671 (G > A) polymorphism i.e. glutamate-to-lysine amino acid substitution at the protein level (also known as Glu504Lys) [16]. The G allele encodes a functional *ALDH2* enzyme needed for aldehyde detoxification, but substituted A allele makes a non-functional isozyme [16]. The fourth selected SNP was from the promotor region of interleukin 6 (*IL-6*) gene and involves a change of guanine to cytosine, at position − 174. The encoded protein is important in inflammation resulting in increased oxidative stress inside the coronary arteries. These SNPs were selected for analysis in Pakistani subjects because 1) Pakistani population represents a unique ethnic group which allows the study of concentrated risk genetic markers 2) the selected SNPs have been reported to modulate serum lipids in Caucasians therefore their analysis in Pakistan can provide the information on the relationship of these genetic variants with lipids and 3) these SNPs have not been previously investigated in the Pakistani population and the current study is the first report of their investigation in our population. We therefore, aimed to genotype them to find their association pattern with CAD in our cohort and to compare the association of single SNPs and their cumulative genetic risk score.

## Methods

For the current study, a total of 623 subjects (219 controls, 404 CAD cases) were recruited. The recruitment, inclusion and exclusion criteria have been described in detail earlier [17]. The patients were angiographically confirmed CAD caes with stenosis of at least one major coronary vessel (50% 0r more of diameter) and diagnosed by cardiologists using biochemical markers like CK-MB, troponin T/I data, echocardiography, ECG, and radiological investigation. The patients were not using lipid-lowering and antihypertensive drugs. Only newly diagnosed cases were included in the study. The controls without any history of CAD at least in first-degree relative were selected [18]. Exclusion criteria for cases were clinical diagnosis of cardiomyopathy, coagulopathy, collagenoses, presence of inflammatory and autoimmune diseases and acute poisoning and for controls were the presence of symptoms of CAD, myocardial infarction (MI), stroke, diabetes mellitus, inflammatory and autoimmune diseases and a familial history of cardiovascular diseases. Serum screening and any infectious blood sample like HIV, hepatitis B and C were not included. Study subjects below 40 years were also excluded. Ethical protocols were strictly followed included all procedures which were in compliance with the declaration of Helsinki and approval was obtained from the institutional ethical committee (Ethical Committee, School of Biological Sciences, University of the Punjab, Lahore, Pakistan).

### Anthropometric measurements

For each study participant gender, age and smoking habit were recorded. The prevalence of comorbidities was noted for the cases and controls. Height (m) and weight (Kg) were measured and body mass index (BMI) in Kg/m^2^ was calculated for every study participant.

### Blood sampling

Venous blood was taken from the median cubital vein by using aseptic technique. 5 ml of blood sample was taken which was divided into two parts. In one part EDTA was added to prevent clotting of blood while the rest was poured in yellow vials with gel clot activator to accelerate blood clotting. The blood in EDTA vials was used for DNA isolation and stored at 4 °C while blood in gel vial was used for obtaining serum to be used for the determination of various biochemical parameters. Serum was separated from blood cells by centrifugation of gel activator vials at 14,000 rpm for 10 min and stored in sterilized eppendorf. Prior to used serum for determination of biochemical parameters, serum was screened for any infectious disease. Serum samples were screened by using a one-step device Accu-chek **®** for hepatitis b **(**HBV), hepatitis c (HCV) and human immunodeficiency virus (HIV) infection.

### Biochemical parameters determination

Serum lipid profile parameters including total cholesterol (TC), triglycerides (TG), low-density lipoprotein cholesterol (LDL-C) and high-density lipoprotein cholesterol (HDLC) were measured by using commercially variable kits (Spectrum Diagnostics, Obour City, Egypt). Determination of all optical density measurements was done by Epoch microplate reader (Biotek Instruments, Highlands Park, USA).

### Genotyping

The salting out manual method was used for the extraction of genomic DNA from the peripheral white blood cells (WBC). The results were analyzed on 1% agarose gel. After isolation, all DNA samples were quantified before amplification using Epoch Biotek micro-plate reader (Biotek Instruments, Highlands Park, USA) and were standardized to the final working concentration of 5 ng/μl was made. For *PONI*, *ITGB3* and *ALDH2* polymorphisms, PCR-RFLP method was employed and for *IL-6* SNP, tetra-ARMS PCR was used. The sequences of the primers used for amplification are as follows: rs662, forward 5’-TATTGTTGCTGTGGGACCTGAG-3′ and reverse 5’-CCTGAGAATCTGAGTAAATCCACT-3′ (product size 238 bp, restriction enzyme, *Alw*I); for rs5918, forward: 5’-GGATTATCCCAGGAAAGACCAC-3′, reverse: 5’-GACTTCCTCCTCAGACCTCCAC-3′ (product size 424 bp, restriction enzyme, *Msp*I); for rs671, forward 5’-CCTGGGCAACAGAGAAAGAT-3′, and reverse 5’-AAACACTGATGGCCTCAAGC-3′ (product size 512 bp, restriction enzyme, *Hin*cII); for rs1800795 forward outer ACCTGGAGACGCCTTGAAGTAACT, reverse outer AAACCAAAGATGTTCTGAACTGAGT, forward inner GCCAGGCAGTCTACAACAGGCC, reverse inner GTGTTCTGGCTCTCCCTGTGTGC (outer product 186 bp, product for C allele 144 bp, product for G allele 86 bp). The PCR mixtures (25 μL) consisted of 1X Taq buffer ([NH4]_2_SO_4_), 2.5 mM MgCl2, dNTP’s mix 200 μM, Taq polymerase (Fermentas) 0.25 U/μl, forward and reverse primers 10 μM and 5 ng/μl DNA template. The PCR program consisted of initial denaturation at 95 °C for 2 min, followed by 35 cycles of denaturation (94 °C for 1 min, annealing at 65 °C for 1 min, extension at 72 °C for 2 min) and a final extension at 72 °C for 5 min. Restriction digestion reaction was optimized for amplified product concentration, duration of digestion and enzyme concentration per reaction volume. The reaction mixture for RFLP (15 μl) consisted of PCR product 10 μl, 10X buffers 1.5 μl, 0.3 U/ μl and 3.2 μl of nuclease-free water. RFLP products were seen by running 2% agarose gel electrophoresis and observing the gel under U.V transilluminator or Gel Doc system.

### Statistical analysis

Microsoft Excel and statistical Package for the Social Sciences (SPSS, IBM statistics version 22) were used for the statistical analysis. Allele and genotype frequencies were calculated for each SNP and the study population was tested for Hardy Weinberg Equilibrium (HWE). The significance of difference of allele and genotype frequencies among cases and controls was checked by chi-squared test. Independent t-test was used to test the difference in mean values of quantitative variables between two groups. Logistic regression analysis was done to check the association of SNPs with the CAD. One way ANOVA (analysis of variance) was used to check the effect of the polymorphisms on lipid profile parameters. GRS was calculated for each study subject by following method: the genotypes were unanimously coded as 0 for homozygous protective genotype, 1 for heterozygous and 2 for homozygous polymorphic genotypes. A summation term was then created by adding the risk allele count for each participant. The mean GRS value of cases and controls was compared by t-test. As four SNPs were included, a subject could have a minimum of 0 and maximum of 8 risk alleles. The GRS was divided into three groups, Group I with risk allele count 0–2, Group II with risk allele count 3–5 and Group III with risk allele count 6–8. A corrected *p*-value less than 0.05 of 0.0125 was used as a statistical cutoff for all tests because of inclusion of four SNPs (0.05/4 = 0.0125).

## Results

### Study subjects’ characteristics

The general characteristics of the study participants have been described in detail elsewhere [17]. The controls had 119 males and 100 females while the cases had 238 males and 166 females. The mean age of the two groups differed significantly, however the controls on the average are of an older age indicating that they have been disease free for a longer time. The prevalence of hypertension and diabetes and smoking was high among patients as compared to the controls. The cases had a more atherogenic lipid profile compared to the controls. The baseline characteristics of the subjects are summarized in Table 1.

**Table 1:** General characteristics of the study subjects

Parameter	Control (219)	Cases (404)	*p*-value
Age	56.0 ± 10.4	59.10 ± 12.64	0.002
Gender M/F	119/100	238/166	–
TC (mg/dL)	175.38 ± 43.0	207.50 ± 53.65	9.5 × 10^−14^
TG (mg/dL)	188.02 ± 66.42	212.12 ± 70.86	3.9 × 10^−5^
LDL (mg/dL)	77.38 ± 20.56	102.85 ± 34.78	1.14 × 10^−21^
HDL (mg/dL)	67.68 ± 17.68	45.14 ± 11.67	1.56 × 10^−65^

Legend: the table compares the general characteristics between the cases and the controls. Values are mentioned as mean ± SD. *P*-values indicate the significance of difference between the two groups as tested by independent sample *t*-test. *TC*Total cholesterol, *TG* Triglycerides, *HDL-c* High Density Lipoprotein cholesterol, *LDL-c* Low Density Lipoprotein cholesterol

### Allele and genotype frequencies of the selected SNPS

The allele/genotype frequencies of all the SNPs are given in Table 2. The minor allele frequency (MAF) for *PON1* polymorphism in controls was 0.425 and in cases, it was 0.491 (OR: 1.15, CI: 0.898–1.470), *p* = 0.02). The MAF for *ITGB3* polymorphism was 0.256 in controls and 0.318 in the cases (OR: 1.179, CI: 0.931–1.477, *p* = 0.05). The MAF for *ALDH2* SNP was 0.326 in the controls and 0.349 in the cases (OR: 0.993, CI: 0.770–1.28, *p* = 0.421). The MAF for the *IL-6* variant was 0.356 in the controls and 0.388 in the cases (OR: 1.21, CI: 0.943–1.540, *p* = 0.153). The MAFs for all SNPs were higher in the cases compared to the controls, however, the difference was statistically insignificant. The logistic regression analysis also revealed a non-significant association of the variants with the CAD.

**Table 2:** Allele and Genotype distribution of the selected SNPs among the cases and the controls

SNP	Genotype/MAF	Controls (*n* = 219)	Cases (*n* = 404)	OR (CI)	*p*-value
rs662 (*PON1*)	AA	84	118	1.15 (0.898–1.470)	0.02
	AG	84	175		
	GG	51	111		
	MAF	0.425	0.491		
rs5918 (*ITGB3*)	CC	129	190	1.179 (0.931–1.477)	0.05
	CT	64	171		
	TT	26	43		
	MAF	0.256	0.318		
rs671 (*ALDH2*)	GG	101	168	0.993 (0.770–1.28)	0.421
	GA	93	190		
	AA	25	46		
	MAF	0.326	0.349		
rs1800795 (*IL-6*)	GG	96	194	1.21 (0.943–1.540)	0.153
	GC	90	133		
	CC	33	99		
	MAF	0.356	0.388		

Legend: Selected SNPs allele/genotype frequencies, *MAF* Minor Allele Frequency, *p*-value indicates the significance of difference in genotype frequencies between cases and controls from chi-squared test, Odds ratios are for risk alleles. *CI* Confidence Interval

### Genetic risk score (GRS) analysis

The GRS was analyzed for descriptive parameters. The minimum GRS in the controls was 0 and in the cases was 1 while maximum GRS in the controls was 6 while in the cases it was 8. The mean GRS was 3.99 ± 1.42 in the controls and 4.29 ± 1.39 in the cases and this difference was statistically significant (*p* = 0.011). The association of GRS with the CAD was checked by logistic regression and was found to be significantly associated (OR: 4.12, CI: 1.003–6.781, *p* = 1.4 × 10^− 4^. The frequency of the subjects in each GRS group was analyzed. There were 66 (10.6%) subjects in group I (GRS = 0–2), 452 (72.6%) subjects in group II (GRS 3–5) and 105 subjects in group III (GRS 6–8).

### Comparison of the effect of individual SNPs and GRS on lipid profile parameters

The effect of individual SNPs across the three genotypes and of GRS across three groups on lipid profile parameters was analyzed and the results are shown in Table 3. The *PON1* SNP rs662 increases TG and decreases HDL-c when the subjects with at least one risk allele are compared to the common homozygotes, however, this effect is not statistically significant whereas the SNP has no effect on TC and LDL-C. The *ITGB3* SNP rs5918 mildly increased TC, TC and LDL-C and decreased HDL-C but the difference was not significant. The *ALDH2* SNP rs671 risk allele increased TC and TG but the effect was insignificant and had no effect on LDL-C and HDL-C. The *IL-6* SNP rs1800795 moderately increased TC, TG and LDL-C and decreased HDL-C, but the effect was still insignificant. When the same analysis was done for the GRS groups, it was clear that the group III with highest GRS had significant effect on all lipid parameters with the strongest association with decrease in HDL-C (*p* = 1.5 × 10^− 3^) followed by increase in TG (*p* = 0.001), LDL-C (*p* = 0.005) and TC (*p* = 0.012).

**Table 3:** Comparison of the effect of genotypes of Singlr SNPs and combined GRS

SNP	Genotype	TC (mg/dL)	TG (mg/dL)	LDL-C (mg/dL)	HDL-C (mg/dL)
rs662 (*PON1*)	AA	175.51 ± 8.47	184.94 ± 7.17	81.38 ± 3.57	70.08 ± 1.77
	AG	170.61 ± 3.75	187.89 ± 6.26	77.05 ± 2.32	68.54 ± 1.68
	GG	181.25 ± 5.08	196.81 ± 13.8	76.45 ± 1.99	65.54 ± 4.05
	*P*	0.242	0.698	0.496	0.357
rs5918 (*ITGB3*)	CC	176.18 ± 6.01	184.68 ± 8.08	76.93 ± 2.61	68.76 ± 2.38
	CT	178.25 ± 4.12	186.02 ± 6.38	77.17 ± 1.93	69.01 ± 1.64
	TT	179.92 ± 5.25	197.79 ± 10.02	78.11 ± 2.91	66.43 ± 2.54
	*P*	0.846	0.549	0.947	0.799
rs671 (*ALDH2*)	GG	170.28 ± 3.74	185.50 ± 11.97	74.33 ± 3.61	64.11 ± 3.26
	GA	175.88 ± 11.76	187.19 ± 6.18	75.88 ± 1.89	76.41 ± 1.86
	AA	180.77 ± 4.61	189.41 ± 7.23	79.37 ± 2.26	64.12 ± 1.72
	*P*	0.218	0.954	0.394	0.492
rs1800795 (*IL-6*)	GG	173.71 ± 7.17	182.02 6.13	72.03 ± 2.52	69.42 ± 3.96
	GC	175.51 ± 3.86	190.54 7.70	76.04 ± 1.99	68.01 ± 1.70
	CC	177.11 ± 5.14	197.06 10.28	80.47 ± 2.37	65.42 ± 1.76
	*P*	0.866	0.478	0.091	0.714
Genetic Risk Score (GRS) groups	Group I (GRS 0–2)	179.95 ± 5.94	196.13 ± 8.89	79.92 ± 3.94	55.41 ± 2.41
	Group II (GRS 3–5)	184.59 ± 2.44	204.08 ± 3.35	85.11 ± 1.54	51.04 ± 1.82
	Group III (GRS 6–8)	193.11 ± 5.49	212.60 ± 8.89	93.41 ± 3.20	49.12 ± 1.71
	*P*	0.012	0.001	0.005	1.5 × 10^−3^

Legend: Values for different lipid parameters are indicated as mean ± S.E Analysis of covariance with age and gender adjusted, *p*-values indicate the association pattern of the selected SNPs with different anthropometric traits

## Discussion

In the current study, we selected a set of four SNPs from different genes known to affect the coronary arteries and genotyped them in a cohort of Pakistani individuals to construct a GRS and investigate whether the use of a GRS can provide better information compared to the individual SNPs. The Pakistani population represents a unique tool to investigate the contribution of genetic markers to diseases based on the restricted religious, social and cultural setting.

The allele and genotype frequencies of the selected SNPs showed that the cases had a higher MAF for all SNPs as compared to the controls, however, except the *PON1* SNP, this difference couldn’t reach statistical significance. This is an indication of the low-modest effect size of the individual variants. At the same time, it must be kept in mind that the SNPs have been genotyped only in a set of participants and the frequencies can be different in the general population therefore, the effect sizes of the SNPs may seem different because of the smaller sample size not actually because they have little role in the CAD progression. These SNPs have previously been reported to be associated with CAD in different populations [19–22].

We showed that the GRS had a significant association with the CAD even when none of the individual variants had a statistically significant association with the disease. This indicated the cumulative power of the GRS over individual SNPs because the risk homozygote frequencies for all SNPs were higher in the cases than the controls but for single SNPs this difference could not achieve statistical significance. However, when GRS of these SNPs was used, the association was very apparent. The GRS between the cases and the controls overlapped and the difference was a bit smaller. This can, to some extent, be attributed to relatively small sample size. However, the difference is still conspicuous and if the number of the SNPs is increased as well as the sample size, the results can be highly applicable.

Regarding the association of individual, similar was the case for the relationship of the individual variants and the GRS with the lipid profile parameters. Individual variants showed mild but insignificant association with the atherogenic profile but the GRS had a highly significant association with the increased TC, TG, LDL-C and decreased HDL-C concentrations. A previous study of Pakistani individuals used the same approach to construct a GRS for 21 variants [23]. This study reported similar findings as reported by us. The current study in combination with the previous study can be used to generate a panel of common variants that can be clinically implemented to calculate a lifetime risk of an individual for developing CAD.

The current study had the limitation of relatively small sample size and inability to include more SNPs. In addition, the more the biochemical parameters included, the more appropriately the mechanism of action of the SNPs could be elucidated. In future, therefore more research with larger sample size, more variants and biochemical parameters should be done on this unique ethnic group so that 1 day a strategy for preventing this fatal condition can be designed.

## Conclusion

In conclusion, a GRS can provide better information for disease association compared to the single SNPs. However, the panel of such SNPs needs to be carefully designed so that the included SNPs are representative of all the candidate and GWAS genes, are of modest effect size and intermediate frequency and have been previously reported to be important in disease predisposition in various ethnicities. This information can then be added to the conventional risk factors so that the high risk individuals can be diagnosed prior to the onset of disease.

## Abbreviations

*ALDH2*: Aldehyde dehydrogenase 2
CAD: Coronary artery disease
CVDs: Cardiovascular diseases
GBD: Global Burden of Disease
GRS: Genetic risk score
HDLC: high-density lipoprotein cholesterol
*IL-6*: Interleukin 6
*ITGB3*: integrin beta 3
LDL-C: Low-density lipoprotein cholesterol
*PON1*: Paraoxanase 1
SNP: Single nucleotide polymorphism
TC: Total cholesterol
TG: Triglycerides
WHO: World Health Organization
